# Is Cardiovascular Regeneration Possible?

**DOI:** 10.14797/mdcvj.1307

**Published:** 2023-11-16

**Authors:** John P. Cooke

**Affiliations:** 1 Houston Methodist DeBakey Heart & Vascular Center, Houston Methodist Hospital, Houston, Texas, US

**Keywords:** stem cell, angiogenesis, cardiomyogenesis, heart failure, peripheral arterial disease

The unequivocal answer is yes…if one is speaking of lower vertebrates. The indomitable zebrafish survives amputation of the apex of its heart, rapidly regenerating a functional myocardium without a scar.^1^ The plucky axolotl is similarly endowed, fully restoring its myocardium after severe cardiac injury.^2^ Whereas fish and amphibians are capable of regenerating myocardium without a scar, this capability has been lost in adult mammals. However, all is not lost. Indeed, it is possible for neonatal mice and pigs to regenerate damaged myocardium without scarring.^3^ Furthermore, scarless healing occurs after fetal surgery in humans.^4^ Even in adult humans, there is a low rate of turnover of cardiomyocytes, equivalent to 0.5% to 1% per year.^5^ Thus, therapeutic cardiac regeneration to repair myocardial damage may be feasible in adult humans. That transformational advance will be facilitated by a greater understanding of the mechanisms by which tissue is restored with such high fidelity in lower vertebrates or young mammals.

On our way toward attaining the goal of therapeutic cardiovascular regeneration, we are learning much about the processes involved in restoration of the heart and vasculature. In the following pages, we review some of these insights and their possible applications. We begin our review with a paper that is based on the foundation laid by Shinya Yamanaka in his Nobel Prize-winning work on induced pluripotency. Dr. Yamanaka galvanized the field of stem cell biology with his discovery of four master regulators of pluripotency (the transcriptional factors Oct 4, Sox2, KLF4, and c-myc).^6^ When these transcriptional factors were overexpressed in a somatic cell, it was transformed into an induced pluripotent stem cell (iPSC) that could be differentiated into any other somatic cell in the body.

Using the Yamanaka method, one can transform skin cells from a patient into iPSCs. It is then possible to coax the iPSCs to differentiate into any cell type, including vascular cells or cardiomyocytes. Such cells could potentially be used therapeutically. For example, iPSC-derived endothelial cells, when implanted into an ischemic limb, improve capillary density and perfusion in a murine model of peripheral arterial disease.^7^ In addition, if the patient has a genetic disorder that is not well characterized, one can generate iPSC-derived cells from that patient to study the disorder in vitro.^8^

Indeed, in this issue Dr. Francisco Altamirano, Leslye Venegas-Zamora, and co-authors describe how human iPSC-derived cardiomyocytes are used to generate engineered heart tissues (EHT) permitting the study of heart failure in vitro. These EHTs have advantages over animal models in that they can be generated with human tissue; the electrophysiological properties of EHTs are similar to those of the human heart, and other cell types derived from the same patient (eg, iPSC-derived endothelial cells and/or fibroblasts) can be added to the EHT to more closely mimic the human heart microenvironment. One can model myocardial ischemic injury, and test new regenerative applications using EHTs.

The lower vertebrates, as well as neonatal mammals, can regenerate the heart without scar, in part because their cardiomyocytes are capable of cell division, a property largely lost in adult mammalian hearts. In this issue, Drs. Daniel Garry, Mary Garry, and colleagues explore the mechanisms, as well as the regenerative applications, of cardiomyocyte proliferation. Clearly, inflammatory signaling is involved in cardiac regeneration, just as it is involved in the generation of iPSCs, as well as other cell fate transitions.^9,10^ Inflammatory signaling increases DNA accessibility to facilitate cell fate changes and similarly may enable the dedifferentiation of cardiomyocytes that is required for their proliferation. In addition, cardiomyocyte replication may be induced by therapeutic manipulation of cardiomyocyte-specific cell cycle regulators, transcriptional factors, growth factors, signaling pathways, and/or metabolic state.

At birth, a metabolic switch occurs from glycolysis to oxidative phosphorylation to support the energy demands of an independent existence. Drs. Hesham Sadek, Ivan Menendez-Montes, and colleagues describe how this change in metabolism also switches off cardiomyocyte proliferation. The mitochondrial metabolism of fatty acids generates reactive oxygen species, which can increase DNA damage leading to the loss of cardiomyocyte proliferation. In addition, the change in metabolism alters the concentration of metabolites that act on the epigenome. For example, glycolysis generates increased levels of uridine diphosphate N-acetylglucosamine (UDP GlycNAC), which is known to post-translationally modify epigenetic proteins to increase DNA accessibility and to support cardiomyocyte proliferation. A variety of drugs or genetic interventions have been used to enhance glycolysis and thereby support cardiomyocyte proliferation and cardiac regeneration in animal models. Such metabolic approaches may be of therapeutic use in the future.

Drs. Yingnan Bai, Zhongyun Xu, and co-authors remind us of the forgotten circulation of lymphatic channels in the heart. This relatively unexplored territory is now yielding some intriguing insights due to the work of Dr. Bai and others. The lymphatic channels in the heart become more prominent after a myocardial infarction to drain the excess fluid, damaged proteins, and inflammatory cells from the injured myocardium. In pre-clinical models, stimulating lymphangiogenesis (eg, with VEGF-C, adrenomedullin, or apelin) can reduce the adverse effects of a coronary occlusion. In related work, Dr. Bai and her colleagues have shown that salt-sensitive hypertension may be in part due to impaired lymphatic clearance of sodium and fluid from the skin. There are intriguing data to support a role for lymphangiogenesis in other cardiac conditions including transplant rejection, heart failure, congenital heart disease, and endocarditis. A more secure characterization of the clinical role of lymphangiogenesis in cardiovascular disease awaits the development of better imaging techniques of the cardiac lymphatic channels in our patients.

Epigenetics is the study of heritable changes in the expression of genes that are not due to changes in DNA sequence. Post-translational modifications of histone proteins, DNA methylation, and interaction of long-noncoding RNA (lncRNA) or small RNAs with genomic DNA or message RNA can alter gene expression by influencing what genes are expressed and to what degree. One of the luminaries in the epigenetics of peripheral arterial disease (PAD), Dr. Zhen Bouman Chen, along with coauthors Naseeb Kaur Malhi, Kevin W. Southerland, and Li Lai, describes how risk factors for cardiovascular disease exert their adverse effects on the vasculature through epigenetic alterations. For example, diabetes mellitus impairs angiogenesis in part by reducing the activation of endothelial nitric oxide synthase by the lncRNA LEENE. Other metabolic changes alter epigenetic regulation of angiogenesis. For example, in the murine model of PAD, ischemia induces a glycolytic shift to increase nuclear acetylcoA. This is the substrate for histone acetylation, which increases DNA accessibility to facilitate cell fate transitions. In this case, the effect of metabolism to increase DNA accessibility facilitates the transdifferentiation of fibroblasts to endothelial cells to support angiogenesis.^11^

Dr. Vihang Narkar continues the theme of vascular regeneration, describing how exercise enhances the vascularization of skeletal muscle. Physical activity activates kinases, transcriptional factors, and nuclear receptors in skeletal muscle to trigger the release of angiogenic factors needed to increase perfusion to the tissue. These effects partially explain the benefits of a supervised exercise program, which is currently the best therapy for improving the symptoms of PAD. The mechanisms by which exercise improves walking distance in patients with PAD are not entirely clear, but improvements in mitochondrial function and perfusion may be involved. The exercise-induced enhancement of perfusion may be due to a combination of arteriogenesis (the positive remodeling of collateral channels), vasculogenesis (the recruitment of circulating angiogenic cells), and angiogenesis (capillary endothelial sprouting), and angiogenic transdifferentiation (as described above, the transformation of resident fibroblasts or other cells into endothelial cells to expand the local microvasculature).^12^ Dr. Narkar points out that a deeper understanding of the mechanisms may lead to the development of an “exercise pill.”

The future for cardiovascular regeneration offers great promise. We have learned much about the mechanisms by which cardiomyocytes and endothelial cells regenerate. Some of these exciting new insights are described in this issue of the *Methodist DeBakey Cardiovascular Journal*. These insights have opened the door to novel therapeutic avenues, some of which are in clinical development. We are most assuredly approaching the day when true cardiac regeneration will be possible for our patients with heart failure and vascular insufficiency.

## Guest Editor Biography

The editors of the *Methodist DeBakey Cardiovascular Journal* express our appreciation to Dr. John P. Cooke for his expertise, insight, and dedication in curating this issue on cardiac regeneration.

## John P. Cooke, MD, PhD

**Figure:**
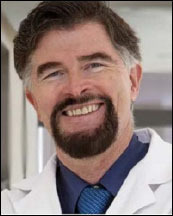


Dr. John P. Cooke is the chair of the Department of Cardiovascular Sciences at the Houston Methodist Research Institute, director of the Center for Cardiovascular Regeneration, and medical director of the RNA Therapeutics Program in the Houston Methodist DeBakey Heart & Vascular Center in Houston, Texas.

He trained in cardiovascular medicine and obtained a PhD in physiology at the Mayo Clinic. He was recruited to Harvard Medical School as an assistant professor of medicine. In 1990, he was recruited to Stanford University to spearhead the program in vascular biology and medicine and was appointed professor in the Division of Cardiovascular Medicine at Stanford University School of Medicine and associate director of the Stanford Cardiovascular Institute until his recruitment to Houston Methodist in 2013.

Dr. Cooke has published over 500 research papers, position papers, reviews, book chapters, and patents in the arena of vascular medicine and biology with over 30,000 citations. He has served on national and international committees that deal with cardiovascular diseases, including the American Heart Association, American College of Cardiology, Society for Vascular Medicine, and the National Heart, Lung and Blood Institute. He has served as president of the Society for Vascular Medicine, as a director of the American Board of Vascular Medicine, and as an associate editor of *Vascular Medicine*.

Dr. Cooke’s research program is focused on vascular regeneration, vascular cell identity, and cell fate. The program is funded by grants from the National Institutes of Health, the American Heart Association, Cancer Prevention Research Institute, and industry.

In his 25 years of translational vascular biology, Dr. Cooke first described and characterized the anti-atherogenic effects of endothelium-derived nitric oxide; the anti-angiogenic effect of the NO synthase inhibitor ADMA; the angiogenic pathway mediated by endothelial nicotinic acetylcholine receptors; the role for this pathway in states of pathological angiogenesis; and developed an antagonist of the pathway that was tested in clinical trials. His clinical research group has explored the use of angiogenic agents and adult stem cells in the treatment of peripheral arterial disease. More recently, his group has generated and characterized vascular cells differentiated from iPSCs of patients with Progeria to understand the role of telomere erosion in this condition of accelerated aging and vascular death. His group’s application of mRNA encoding human telomerase to reverse aging in this condition and other age-related diseases is promising.

## References

[1] Poss KD, Wilson LG, Keating MT. Heart regeneration in zebrafish. Science. 2002 Dec 13;298(5601):2188-90. doi: 10.1126/science.1077857 12481136

[2] Becker RO, Chapin S, Sherry R. Regeneration of the ventricular myocardium in amphibians. Nature. 1974 Mar 8;248(5444):145-7. doi: 10.1038/248145a0 4818918

[3] Porrello ER, Mahmoud AI, Simpson E, et al. Transient regenerative potential of the neonatal mouse heart. Science. 2011 Feb 25;331(6020):1078-80. doi: 10.1126/science.1200708 21350179 PMC3099478

[4] Moore AL, Marshall CD, Barnes LA, Murphy MP, Ransom RC, Longaker MT. Scarless wound healing: Transitioning from fetal research to regenerative healing. Wiley Interdiscip Rev Dev Biol. 2018 Mar;7(2):10.1002/wdev.309. doi: 10.1002/wdev.309 PMC6485243 29316315

[5] Bergmann O, Bhardwaj RD, Bernard S, et al. Evidence for cardiomyocyte renewal in humans. Science. 2009 Apr 3;324(5923):98-102. doi: 10.1126/science.1164680 19342590 PMC2991140

[6] Takahashi K, Yamanaka S. Induction of pluripotent stem cells from mouse embryonic and adult fibroblast cultures by defined factors. Cell. 2006 Aug 25;126(4):663-76. doi: 10.1016/j.cell.2006.07.024 16904174

[7] Rufaihah AJ, Huang NF, Jamé S, et al. Endothelial cells derived from human iPSCS increase capillary density and improve perfusion in a mouse model of peripheral arterial disease. Arterioscler Thromb Vasc Biol. 2011 Nov;31(11):e72-9. doi: 10.1161/ATVBAHA.111.230938 21836062 PMC3210551

[8] Mojiri A, Walther BK, Jiang C, et al. Telomerase therapy reverses vascular senescence and extends lifespan in progeria mice. Eur Heart J. 2021 Nov 7;42(42):4352-4369. doi: 10.1093/eurheartj/ehab547 34389865 PMC8603239

[9] Lee J, Sayed N, Hunter A, et al. Activation of innate immunity is required for efficient nuclear reprogramming. Cell. 2012 Oct 26;151(3):547-58. doi: 10.1016/j.cell.2012.09.034 23101625 PMC3506423

[10] Meng S, Zhou G, Gu Q, Chanda PK, Ospino F, Cooke JP. Transdifferentiation Requires iNOS Activation: Role of RING1A S-Nitrosylation. Circ Res. 2016 Oct 14;119(9):e129-e138. doi: 10.1161/CIRCRESAHA.116.308263 27623813 PMC5065398

[11] Lai L, Reineke E, Hamilton DJ, Cooke JP. Glycolytic Switch Is Required for Transdifferentiation to Endothelial Lineage. Circulation. 2019 Jan 2;139(1):119-133. doi: 10.1161/CIRCULATIONAHA.118.035741 30586707 PMC6311718

[12] Meng S, Lv J, Chanda PK, Owusu I, Chen K, Cooke JP. Reservoir of Fibroblasts Promotes Recovery From Limb Ischemia. Circulation. 2020 Oct 27;142(17):1647-1662. doi: 10.1161/CIRCULATIONAHA.120.046872 32820662 PMC7987209

